# Descriptive epidemiology of soft tissue sarcomas and gastrointestinal stromal tumors in Thailand

**DOI:** 10.1038/s41598-022-15711-8

**Published:** 2022-07-27

**Authors:** Jeerawan Klangjorhor, Donsuk Pongnikorn, Pattaralawan Sittiju, Areerak Phanphaisarn, Parunya Chaiyawat, Pimpisa Teeyakasem, Patiwat Kongdang, Sutpirat Moonmuang, Narate Waisri, Karnchana Daoprasert, Taweechok Wisanuyotin, Chalongpon Santong, Siriphon Sitthikong, Pakjai Tuntarattanapong, Paradee Prechawittayakul, Dumnoensun Pruksakorn

**Affiliations:** 1 Musculoskeletal Science and Translational Research (MSTR) Center, Department of Orthopedics, Faculty of Medicine, Chiang Mai University, Chiang Mai, Thailand; 2 Cancer Registry Unit, Lampang Cancer Hospital, Lampang, Thailand; 3 Thailand Excellence Center for Tissue Engineering and Stem Cells, Department of Biochemistry, Faculty of Medicine, Chiang Mai University, Chiang Mai, Thailand; 4 Chiang Mai Cancer Registry, Faculty of Medicine, Chiang Mai University, Chiang Mai, Thailand; 5 Department of Orthopaedic, Faculty of Medicine, Khon Kaen University, Khon Kean, Thailand; 6 Cancer Unit, Srinagarind Hospital, Faculty of Medicine, Khon Kaen University, Khon Kean, Thailand; 7 Cancer Registry Unit, National Cancer Institute, Bangkok, Thailand; 8 Department of Orthopaedic, Faculty of Medicine, Prince of Songkla University, Songkhla, Thailand; 9 Cancer Information Center, Faculty of Medicine, Prince of Songkla University, Songkhla, Thailand; 10 Center of Multidisciplinary Technology for Advanced Medicine (CMUTEAM), Faculty of Medicine, Chiang Mai University, Chiang Mai, Thailand

**Keywords:** Cancer, Health care, Risk factors

## Abstract

This study aimed to analyze burden of STS and GIST in population and survival rate which represented the current situation of treatment in Thailand. The data was collected from five population-based cancer registries around the country for the period 2001 through 2015. The Segi world standard population was used to calculated age-standardized incidence rates (ASR). Standardized rate ratios (SRR) were used to compare populations. Joinpoint Trend Analysis was used to assess changes in incidence. STATA was used to examine patient survival rates. During the study period, 4080 cases of STS and 457 cases of GIST were reported. The ASR of STS and GIST was 2.14/100,000 person-years and 0.22/100,000 person-years, respectively. The most common histological types of STS were unspecified sarcoma (24.8%), leiomyosarcoma (19.0%) and liposarcoma (11.4%). The overall ASR of STS in Thailand was relatively low compared to Western countries. The five-year survival rate was 62.6% for STS and 63.4% for GIST, which was comparable to the rates reported in other countries. This is the first report of STS and GIST from PBCRs in Thailand. Based on current healthcare service, an overall survival rates of STS and GIST are comparable to those reported from others.

## Introduction

Sarcomas are rare malignant mesenchymal neoplasms, accounting for 1% of all malignant tumors^1^. Radiology divides sarcomas into two types: bone sarcomas (dense tissue-like sarcomas that partially blocks X-rays) and soft tissue sarcomas (soft tissue which allows X-rays to pass easily). The American Cancer Society has stated that soft tissue sarcoma (STS) can occur in connective and soft tissues in any part of the body as well as in visceral organs^2^. Visceral sarcomas are not currently classified as STS by the World Health Organization (WHO)^1^ although gastrointestinal stromal tumors (GIST) are included in the 2013 WHO classification of STS^3,4^. Whereas, the most studies report the combined incidence of STS and visceral sarcoma, but report GIST incidence separately. For worldwide comparisons, the latter criteria were used to report in this study.

Most population-based studies on STS incidence have been undertaken in Western countries^5–9^ and include only limited information on the Asian countries^10,11^. The actual overall incidence of STS in each histological type is still uncertain since to the difficulty in determining a definite diagnosis related to histological heterogeneities and the lack of specific molecular identification. In comprehensive reviews of Western countries, STS has been estimated to account for 0.7–1% and 4–8% of all malignant tumors in adults and children, respectively^12,13^. For Thailand, three population-based cancer registry (PBCR) reports covering the periods 1988–1991^14^, 1995–1997^15^, and 2003–2005^16^ present the incidence of childhood cancer at the national level. From these reports, STS accounted for 4.2–4.9% of all malignant tumors in children. The incidence and survival rates of childhood STS have been reported for the period 1985–2010 in Khon Kaen, a province in northeastern Thailand^17^. That report showed rhabdomyosarcoma and fibrosarcoma to be the most common histological types of STS in children. That incidence rate is relatively low when compared to the rate in other Asian and Western countries, but the age-related patterns of rhabdomyosarcoma and fibrosarcoma were similar to those seen elsewhere^14,15^. The overall survival rate for STS in children in Khon Kaen was lower than in developed countries^16,17^. Most studies in Thailand are case reports or clinic-pathological data analyses from individual hospitals and mainly focus on organ-specific sarcomas or groups of tumors and report on both carcinomas and sarcomas. Tumors of female reproductive organs are the most frequently presented in those reports. To date, no epidemiology research reports focusing exclusively on STS in Thailand have been published.

This study is the first report of the incidence rate of STS and GIST in Thailand. Population-based cancer registry data for the years 2001 to 2015 was obtained from five PBCRs located in different regions of the country. In addition to gender, the incidence rate and survival rate of STS, as well as specific histological types and primary sites were recorded. This information is crucial in evaluating the overall quality of healthcare services in the country as well as in identifying gaps of improvements in medical care.

## Results

### Demographic data

Supplementary Figure S1A shows the geographic location of the 5 PBCRs in 5 Thai provinces. In 2015, Chiang Mai, the largest province in northern region and fifth largest in Thailand, had approximately 1.6 million people. With a population of 0.7 million people, Lampang, is the third largest province in the north (thirty-third largest in Thailand). Khon Kaen, the second largest province in the northeast and fourth largest in Thailand, had a population of over 1.7 million people. Bangkok, Thailand’s capital and largest city, had a population of around 5.5 million people. Songkhla, the third largest province in the country in area and second largest in the southern region, had 1.4 million inhabitants (eleventh most populous in the nation). These five provinces had a combined total of 11 million people, accounting for 16.9% of Thailand’s population. Because of a decline in the birth rate, in part due to Thailand’s National Family Planning Policy, Thailand’s population structure has undergone a “pyramid-to-pillar transition since 1970. Together with an increased life expectancy, the demographic structure of each of these provinces has been of a constrictive type during study period (Supplementary Fig. S1B).

### Data quality

A total of 4080 cases of STS and 457 cases of GIST were identified and recorded in the five PBCRs registries during the study period (Supplementary Table S1). The average percentage of cases of STS diagnosed by morphological verification (MV) was 99.7% (range 99.1–100.0%) and of GIST was 99.4% (range 98.2–100.0%). No cases were verified by death certificate only (DCO). For STS, verification of 12 cases (0.3%) involved histological and physical examinations, 5 cases (0.1%) used endoscopy and radiology, and 2 cases (0.05%) used surgery and autopsy without histological examination. Of these 19 cases, 14 were from the Bangkok area (0.6%), 1 case from Chiang Mai (0.1%) and 4 cases from Khon Kaen (0.9%). For GIST (Supplementary Table S2), 3 cases (0.7%) were verified using histology and physical examination, while 2 cases (0.4%) were diagnosed using endoscopy and radiology: four from Bangkok (0.2%) and one from Khon Kaen (0.2%). Morphological verification was present in more than 96% of cases in each histological type. The lowest percentage morphologically verified cases (%MV) was extraskeletal Ewing sarcoma (98.0%).

### Incidence of STS and GIST in Thailand

Crude incidence rates and age-standardized incidence rates were categorized by histology type and registry (Table 1). Total crude incidence was 2.51/100,000 person-years for STS and 0.28/100,000 person-years for GIST, with a combined annual average for the 5 PBCRs of 272 cases of STS and 30 cases of GIST. The estimated number of new cases per year in the country overall was 1604 for STS and 180 for GIST.

**Table 1:** Crude and age-standardized incidence of soft tissue sarcoma and gastrointestinal stromal tumor by histological subtype and sex. Case count, crude and age-standardized incidence rates per 100,000 person-years, and SRR are reported. The ASR was adjusted to the Segi world standard population. * indicates that the rates are significantly different at the 5% level (*p < 0.05*).

Histological Subtype	ICD-O-3	All			Male			Female			SRR (95% CI) Male/Female
		***n***	***Crude***	***ASR***	***n***	***Crude***	***ASR***	***n***	***Crude***	***ASR***	
All STS		4537	2.79	2.36	1857	2.37	2.11	2680	3.18	2.59	0.81 (0.64–1.03)
STS without GIST		4080	2.51	2.14	1634	2.08	1.87	2446	2.91	2.38	0.79 (0.61–1.01)
Sarcoma, NOS	8800–8806, 8830	1013	0.62	0.52	518	0.66	0.58	495	0.59	0.46	1.25 (0.76–2.05)
UPS (MFH)	8830, 8802	379	0.23	0.19	201	0.26	0.22	178	0.21	0.16	1.39 (0.63–3.10)
Leiomyosarcoma/ Myosarcoma	8890, 8894–8896	775	0.48	0.39	206	0.26	0.22	569	0.68	0.53	0.42 (0.23–0.76)*
Liposarcoma	8850–8855, 8857–8858	464	0.28	0.23	252	0.32	0.27	212	0.25	0.19	1.42 (0.68–2.98)
Dermatofibrosarcoma	8832, 8833	263	0.16	0.14	130	0.17	0.14	133	0.16	0.13	1.08 (0.39–3.01)
Rhabdomyosarcoma	8900–8902, 8910, 8912, 8920	241	0.15	0.18	118	0.15	0.18	123	0.15	0.17	1.06 (0.38–2.97)
Mixed tumor	8940, 8950–8951	213	0.13	0.12	22	0.03	0.03	191	0.23	0.19	0.16 (0.05–0.53)*
Phyllodes tumor	9020	178	0.11	0.08	2	< 0.01	< 0.01	176	0.21	0.15	0.01 (0.00–0.25)*
Vascular tumors	9120, 9130, 9133	147	0.09	0.07	90	0.12	0.10	57	0.07	0.05	1.99 (0.54–7.35)
Stromal sarcoma	8930, 8931, 8933, 8935	155	0.10	0.07	1	< 0.01	< 0.01	154	0.18	0.14	0.01 (0.00–0.12)*
Fibromatous neoplasms	8810–8815, 9150	176	0.11	0.09	87	0.11	0.10	89	0.11	0.09	1.09 (0.33–3.60)
Synovial sarcoma	9040–9041, 9043–9044	134	0.08	0.07	67	0.09	0.08	67	0.08	0.07	1.14 (0.31–1.23)
MPNST	9540, 9560, 9561	165	0.10	0.09	68	0.09	0.07	97	0.12	0.10	0.70 (0.21–2.38)
Extraskeletal Ewing sarcoma	9260, 9364	50	0.03	0.04	29	0.04	0.04	21	0.02	0.03	1.34 (0.13–13.89)
Extra-renal rhabdoid tumor	8963, 8964	19	0.01	0.02	11	0.01	0.02	8	0.01	0.01	2.00 (0.04–93.29)
Granular cell tumor	9580	18	0.01	0.01	4	0.01	< 0.01	14	0.02	0.01	0.38 (0.01–15.58)
Alveolar soft part sarcoma	9581	17	0.01	0.01	8	0.01	0.01	9	0.01	0.01	0.82 (0.02–40.56)
Phosphaturic mesenchymal tumor	8990, 8991	13	0.01	0.01	6	0.01	0.01	7	0.01	0.01	0.64 (0.01–63.85)
Extraskeletal Chondrosarcoma	9231,9240	12	0.01	0.01	5	0.01	0.01	7	0.01	0.01	1.00 (Undefined)
Chordoma	9370, 9371	11	0.01	0.01	3	< 0.01	< 0.01	8	0.01	0.01	0.50 (0.00–98.54)
Myxosarcoma	8840	7	< 0.01	< 0.01	2	< 0.01	< 0.01	5	0.01	< 0.01	0.40 (0.00–113.44)
Perineurioma	9571	4	< 0.01	< 0.01	3	< 0.01	< 0.01	1	< 0.01	< 0.01	3.00 (0.00–10,916.80)
Giant cell sarcoma of soft part	9251	3	< 0.01	< 0.01	2	< 0.01	< 0.01	1	< 0.01	< 0.01	0.40 (0.00–113.44)
Myofibroblastic sarcoma	8825	2	< 0.01	< 0.01	0	0	0	2	< 0.01	< 0.01	Undefined
STS without Phyllodes tumor		4359	2.68	2.28	1855	2.37	2.11	2504	2.98	2.43	0.87 (0.68–1.11)
STS without GIST and Phyllodes tumor		3902	2.40	2.07	1632	2.08	1.87	2270	2.70	2.23	0.84 (0.65–1.08)
GIST	8936, 8891	457	0.28	0.22	223	0.28	0.24	234	0.28	0.20	1.23 (0.55–2.47)

The ASRs of STS were highest in Chiang Mai and Bangkok (2.61 and 2.39/100,000 person-years, respectively), lower in Songkhla (1.81/100,000 person-years) and lowest in Lampang and Khon Kaen (1.55 and 1.54/100,000 person-years, respectively). The ASRs of GIST from Chiang Mai and Bangkok were also the highest among the cancer registries (0.25/100,000 person-years), followed by Khon Kaen (0.18/100,000 person-years), Lampang (0.13/100,000 person-years), and Songkhla (0.05/100,000 person-years).

### Type and subtype-specific rates of STS

The seven most common histological types combined included 77.1% of the STS cases. The first rank was unspecified sarcoma, accounting for 24.8% of STS registrations. This was followed by leiomyosarcoma (19.0%), liposarcoma (11.4%), dermatofibrosarcoma (6.4%), rhabdomyosarcoma (5.9%), mixed tumors (5.2%) and phyllodes tumor (4.4%). When GIST was included, it was the fourth most common sarcoma (10.1%) following unspecified sarcoma, leiomyosarcoma and liposarcoma.

According to previous epidemiologic research, liposarcoma and rhabdomyosarcoma are not a single disease entity since they have different histological features and age-specific distribution patterns. As to liposarcoma, approximately half the cases were unspecified liposarcoma (47.0%). Among the other common histological subtypes were myxoid (23.3%), well differentiated (11.2%), pleomorphic (7.3%) and dedifferentiated (5.6%). For rhabdomyosarcoma, the most common subtypes were unspecified rhabdomyosarcoma (51.9%), followed by embryonal (27.8%), alveolar (10.0%) and pleomorphic (8.3%). Mixed type and spindle cell were very rare, accounting for 1.2% and 0.8% of all rhabdomyosarcomas.

### Predominance of STS in females

Soft tissue sarcoma overall had a slightly higher ASR in females (2.38/100,000 person-years) than in males (1.87/100,000 person-years) (Table 1). The overall male–to–female ratio of STS was 0.79:1 (95% CI 0.61–1.01). This difference was due to histological types with a significant female predominance, including leiomyosarcoma (0.42:1, 95% CI 0.23–0.76), mixed tumor (0.16:1, 95% CI 0.05–0.53), phyllodes tumor (0.01:1, 95% CI 0.00–0.25) and stromal sarcoma (0.01:1, 95% CI 0.00–0.12). Additionally, the rates of females were 0.70/100,000 person-years (18.2%) for sarcoma of female genital organs and 0.21/100,000 person-years (5.9%) for sarcoma of the breast, whereas sarcoma of male genital organs had a rate of only 0.04/100,000 person-years (0.8%). For the remaining common histological types of STS, males had rates slightly higher than those in females. No sex predilection was found for GIST.

### Site-specific rates of STS and GIST

Approximately half of STS arose in connective, subcutaneous and other soft tissues (Supplementary Table S3), which imply locations in the extremities and trunk (45.7%). Unspecified sarcoma (67.5%), liposarcoma (74.4), UPS (79.9%), rhabdomyosarcoma (56.4%), vascular tumors (37.4%), fibrosarcoma (77.8%) and synovial sarcoma (88.1%) presented predominantly in these locations as well (Supplementary Table S4). The second most common location of STS was on female genital organs (18.3%) (Supplementary Table S3). Leiomyosarcoma was the predominant STS type on this location (Supplementary Table S4). Other common locations of STS were skin (6.9%), breasts (6.0%) and digestive organs (5.8%). The most common primary sites of GIST were the stomach (45.7%) and small intestine (24.7%) (Supplementary Table S3).

### Age-specific incidence rates

Seven and one tenth percent of STS arose in children and adolescents (age 0–19 years), while 78% of the cases were in adults (age 20–69 years) and 14.8% were in the elderly (70 years and over) (Supplementary Table S1). Only 1.1% of GIST patients were found in children and adolescents, while 75.7% of GIST occurred in adults and 23.2% were found in the elderly (Supplementary Table S2).

**Figure 1:**
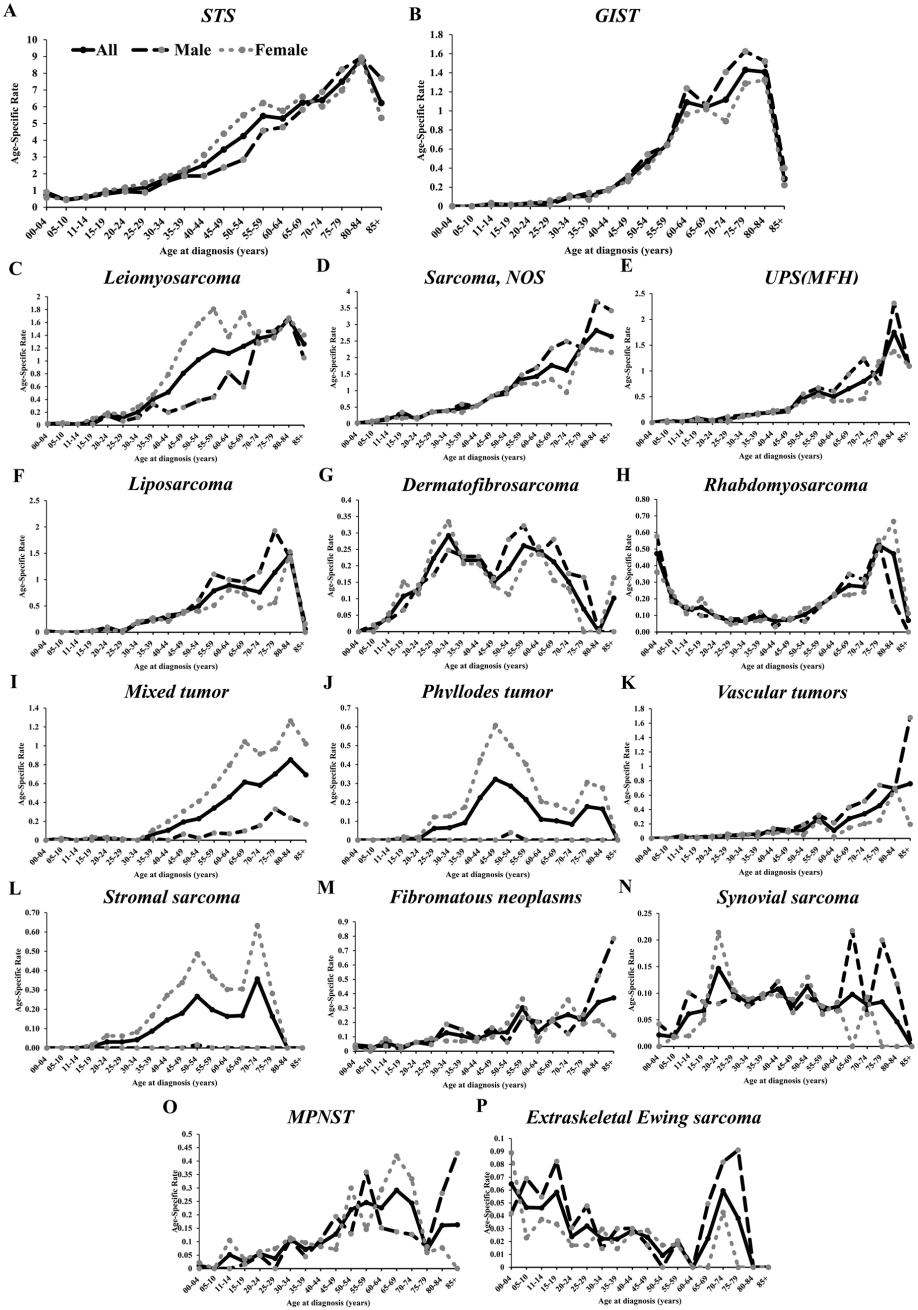
Age-specific incidence rates of STS and GIST by major histological type and sex. *STS* soft tissue sarcoma, *GIST* gastrointestinal stromal tumor, *NOS* not otherwise specified, *UPS* Undifferentiated pleomorphic sarcoma, *MFH* malignant fibrous histiocytoma, *MPNST* malignant peripheral nerve sheath tumors.

Age-specific incidence rates of STS overall and categorized by histological type and GIST are shown in Fig. 1. The overall incidence rate of STS and GIST showed an increase with age, peaking at 80–84 years for STS (Fig. 1A) and 75–79 years for GIST (Fig. 1B). The majority of STS histological types showed an increasing rate with age (Fig. 1D–F), similar to the overall STS trend, although some types of STS showed distinctly different age distribution patterns. For example, dermatofibrosarcoma had a high incidence rate over a wide age range (25 to 69) (Fig. 1G). Phyllodes tumor showed a peak incidence in females aged 40–55 years, i.e., during the perimenopausal period (Fig. 1J). Synovial sarcoma and extraskeletal Ewing sarcoma were found to be distributed over an age range of 5 to 85 years (Fig. 1N,P). The incidence of rhabdomyosarcoma showed a bimodal pattern, peaking during childhood (0–4 years) and older adults (55 +) (Fig. 1H). Age-specific rates of rhabdomyosarcoma categorized by cell type showed significant age variation. Rhabdomyosarcoma NOS and alveolar subtypes had a bimodal pattern, but with the highest incidence in older adults (Supplementary Fig. S2A and B) while embryonal rhabdomyosarcoma had a peak incidence at age 0–4 (Supplementary Fig. S2C). Stromal sarcoma (Fig. 1L) and MPNST (Fig. 1O) showed increasing incidence in the middle age group.

### Time trends of incidence rates

The time trends of incidence of STS and GIST overall and by histological type during 2001–2015 are presented in Supplementary Figure S3. The overall ASRs of STS were relatively stable during the study period (Supplementary Fig. S3A), while the ASR of GIST increased significantly at an average of 8.6% per year (95% CI 5.0–12.2) (Supplementary Fig. S3B). The incidence trends of STS subtypes variously exhibited increasing, declining and stable rates. There were slightly declining trends in the incidence of leiomyosarcoma (Supplementary Fig. S3C), UPS (Supplementary Fig. S3E), rhabdomyosarcoma (Supplementary Fig. S3H), mixed tumor (Supplementary Fig. S3I), vascular tumors (Supplementary Fig. S3K) and extraskeletal Ewing sarcoma (Supplementary Fig. S3P) while the incidence of dermatofibrosarcoma (Supplementary Fig. S3G) and synovial sarcoma (Supplementary Fig. S3N) significantly increased. Unspecified sarcoma (Supplementary Fig. S3D), Liposarcoma (Supplementary Fig. S3E) and MPNST (Supplementary Fig. S3O) showed a slightly increasing trend.

### Survival rates

**Figure 2:**
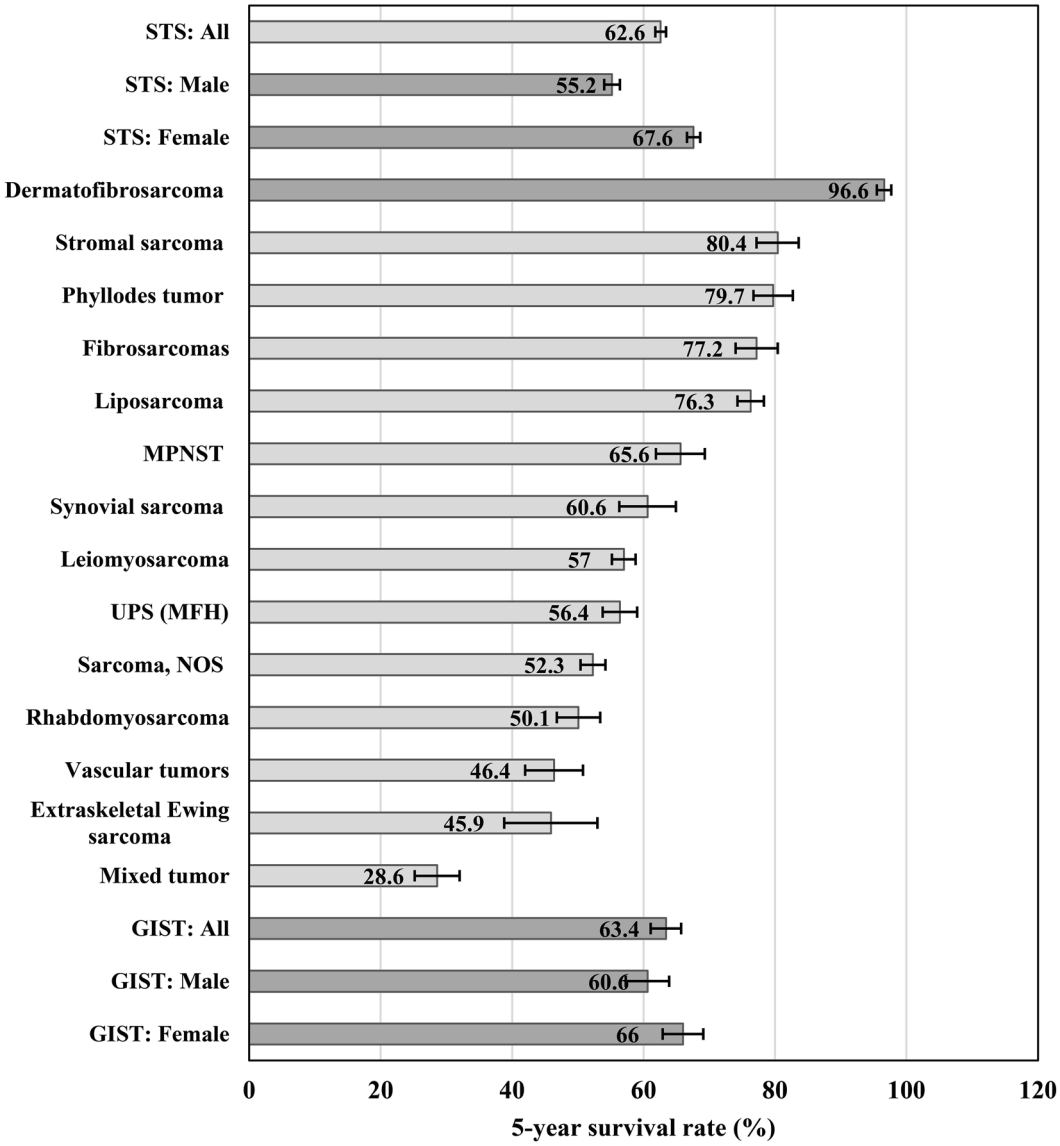
Five-year relative survival rate of STS and GIST by sex and histological type. *STS* soft tissue sarcoma, *GIST* gastrointestinal stromal tumor, *NOS* not otherwise specified, *UPS* Undifferentiated pleomorphic sarcoma, *MFH* malignant fibrous histiocytoma, *MPNST* malignant peripheral nerve sheath tumors.

The patients in this cohort had follow-up time ranging from 0 to 231.3 months, with a median survival time of 75.6 months. The 5-year survival for STS by histological type and GIST (Fig. 2) and for STS by primary site (Supplementary Fig. S4) were reported. The five-year survival rate was 62.6% for STS overall and 63.4% for GIST. For both STS and GIST, the survival rates in males were slightly lower than those in females. Dermatofibrosarcoma had the highest survival rate (96.6%), which is consistent with the rate of STS of the skin (92.1%), the most common primary site of this subtype. The survival rate of phyllodes tumor was 79.7%, which is also consistent with the survival rate of its most common primary site, the breast (78.5%). The major subtypes of STS, leiomyosarcoma and unspecified sarcoma, had 5-year survival rates of 57% and 52.3%, respectively. Survival rates of leiomyosarcoma varied among the most frequent primary sites: 44.6% for female genital organs, 62.1% for connective, subcutaneous and other soft tissues, 64.7% for retroperitoneum and peritoneum and 78.2% for digestive organs. Mixed tumors had the lowest 5-year survival rate at only 28.6%. Soft tissue sarcoma of the female genital organs, thyroid and endocrine glands and urinary tract, where the main subtype was leiomyosarcoma, had survival rates of less than 50%.

## Discussion

The incidence of STS and GIST in Thailand was analyzed using data from 5 PBCRs located in different geographic regions of the country. To avoid selection bias, a population-based dataset was obtained using precise and uniform validation procedures. During 2001–2015, the majority of cases of STS and GIST recoded in these PBCRs had been confirmed by morphological verification, affirming the accuracy of the reports used in this study. This study represents the first complete descriptive overview of the epidemiology of STS and GIST in Thailand.

The age-standardized overall incidence rate of STS in Thailand during 2001–2015 (2.14/100,000 person-years) was close to that in Japan (2.0/100,000 person-years)^10^, but relatively low compared to Taiwan (3.8/100,000 person-years)^11^ and Western countries (4.2–5.8/100,000 person-years)^5–9^. The descriptive ASR reports shown in Table 2 reveal a low incidence of STS in Asians. There is, however, a paucity of data on racial disparities in this tumor type. According to a National Cancer Institute SEER program report on STS incidence rates from 1973 to 2008, African Americans (Blacks) had the highest overall incidence rate (5.1/100,000 person-years), followed by Caucasians (Whites) (4.5/100,000 person-years), while American Indians as well as Asians and Pacific Islanders had the lowest rate (2.8/100,000 person-years)^18^. Other studies from SEER also reported that African Americans had a higher incidence rate than Caucasians^5,19^. These data indicated that race might have an impact on the incidence of STS. To get a better understanding of the possible processes behind these racial disparities, more research on the biologic and genetic differences in sarcomas by race and ethnicity is needed.

**Table 2:** Age-standardized incidence rates of soft tissue sarcoma by major histological type and gastrointestinal stromal sarcoma in Thailand and from comparable registries in other countries. The ASR was adjusted to the Segi world standard population.

Country (period of study)	ASR (per 100,000 person-years)					
	STS	GIST	Leiomyosarcoma	Sarcoma, NOS	Liposarcoma	UPS
Thailand (2001–2015) *Current study	2.14	0.22	0.39	0.52	0.23	0.19
Osaka, Japan (1978–2007)^10^	2.0	–	–	–	–	–
Taiwan (2007–2013)^11^	3.8	1.55	0.53	0.65	0.63	0.19
SEER program of United States (1978–2001)^5^	5.03	–	1.23	0.65	0.59	0.88
RARECARE program of Europe (1995–2002)^6^	4.2	0.1	–	–	–	–
France (2005–2007)^7^	4.2	0.7	0.5	0.7	0.7	–
Germany (2005–2012)^8^	4.5	–	–	–	–	–
Europe (2007–2008)^9^	5.76	1.36	0.93	0.35	0.99	0.37

In our study, STS had a slightly higher incidence in females and this characteristic was due to histological types with a significant female predominance, i.e., leiomyosarcoma, stromal sarcoma, and mixed tumors that were predominantly located in female genital organs, especially in the uterus, and phyllodes tumor, which specifically arose in the breast. The age-specific incidence rates of organ-specific sarcomas (Supplementary Figure S5) showed that the rate of uterine leiomyosarcoma, endometrial stromal sarcoma, and phyllodes tumor increased rapidly throughout the childbearing years and peaked at the perimenopausal age of 45 to 54 years. Then, during the menopausal phase, the rates plateaued or declined. The rates of mixed tumors that originated in the uterus showed a slight delay, but the same pattern as the others. Our findings are consistent with previous studies that found the pattern to be similar to female breast cancer age-specific incidence curves, implying that sex hormones may be involved^5,9^. There is the study reported that estrogen, progesterone and androgen receptors are expressed in 70–80% of uterine leiomyosarcomas^20^. These data support the evidence of estrogen-related cancers, which involves initiation and promoting the process of carcinogenesis by enhancing the rate of cell division and inducing gene mutation.

Overall, trends of STS were found to be consistent in our cohort during this study period, including both increases and decreases in incidence among histological types. It is still unclear whether the trends of sarcoma and its histological types are related to environmental and/or behavioral changes or whether they are due to changes in the registration procedure. The World Health Organization (WHO) classification of tumors of soft tissue and bone was updated in 2002^1^. However, the new 2013 edition includes many changes in classification, mostly the result of novel genetic findings in different histological types^21^. These classification changes which occurred during the period of this study could possibly have influenced the incidence rates and trends of STS reported in this study, e.g., the decrease in the incidence of leiomyosarcoma and the increase in the incidence of GIST. In this case, the increase in the frequency of GIST could be due in part to the 2013 reclassification of numerous mesenchymal gastrointestinal tumors previously classified as leiomyosarcomas. Additionally, the increased incidence of GIST is related to the introduction in 2001 of CD117 antigen detection using immunohistochemical staining^22^.

There have also been significant changes in the classification of MFH. Consequently, the decreased incidence of MFH is probably due, at least in part, to changes in diagnostic criteria which reclassified some MFH sarcomas as UPS (Supplementary Fig. S6A) or pleomorphic varieties of other sarcomas. These findings suggest that advances in molecular biology have been critical in the identification of sarcoma subtypes and the refinement of sarcoma classification. The molecular technique can be used with approximately half of all sarcomas with a specific known genetic abnormality^7,23^. Furthermore, in some subtypes of sarcoma, molecular characterization already has clinical implications including disease prognosis, therapy selection, and treatment response.

Reclassification according to the WHO 2013 resulted in the change of incidences in our study after the guideline was proposed. Beside leiomyosarcoma and GIST, neurilemoma (ICD-O-3 code: 9560/3) was changed to MPNST (WHO 2013 code: 9540/3), this classification changes significantly altered the incidence through the study period. There are the changes that altered the incidence after the WHO 2013 classification was published, i.e., round cell liposarcoma and mixed liposarcoma (ICD-O-3 code: 8853/3 and 8855/3) changed to round cell/ myxoid liposarcoma (WHO 2013 code: 8852/3) (Supplementary Fig. S6B), hemagiopericytoma (ICD-O-3 code: 9150/3) changed to solitary fibrous tumor (WHO 2013 code: 8815/3) (Supplementary Fig. S6C), and Ewing sarcoma (ICD-O-3 code: 9260/3) changed to extraskeletal Ewing sarcoma (WHO 2013 code: 9364/3) (Supplementary Fig. S6D). Incidence of the obsolete ICD-O-3 morphological codes were decrease and their alignment codes were increase especially in 2015. This indicates that the practices in our country follow the updated standard guidelines. However, there is the change that not alter the rate, i.e., fibroblastic liposarcoma (ICD-O-3 code: 8857/3) changed to well differentiated/dedifferentiated liposarcoma (WHO 2013 code: 8851/3 and 8858/3) (Supplementary Fig. S6E). This finding could be due to the extremely low rate of obsolete codes, which has no bearing on the rate of alignment codes.

Moreover, the behavior of some morphology has been changed from "malignant" in ICD-O-3 to "borderline" in WHO 2013, i.e., well differentiated liposarcoma ICD-O-3 code: 8851/3 changed to WHO 2013 code: 8850/1 (Supplementary Fig. S7A), dermatofibrosarcoma protuberans ICD-O-3 code: 8832/3 changed to WHO 2013 code: 8832/1, and pigmented dermatofibrosarcoma protuberans ICD-O-3 code: 8833/3 changed to WHO 2013 code: 8833/1 (Supplementary Fig. S7B). From these changes, the decreased incidence of these morphological codes was observed in 2015. In this study, we found that dermatofibrosarcoma had the highest 5-year survival rate (96.6%). This observation supports the change of behavior from malignant to borderline in this sarcoma group. This indicates that the practices in our country follow the updated standard guidelines.

The significant differences in sarcoma incidence patterns by histologic type and subtypes supports the concept that these tumors are etiologically unique, as noted in previous studies^5,7^. That means in studies of potential risk factors they should be considered separately. In our cohort, rhabdomyosarcoma had a bimodal distribution of age-specific rates dependent on histological subtypes, a finding similar to most previous studies. The fact that rhabdomyosarcoma is not a single disease entity was highlighted by this observation. That means modern diagnosis techniques focusing on genetic alterations could result in more precise definition and identification of rhabdomyosarcoma subtypes^24^.

The survival rates of STS and GIST patients in Thailand were comparable to the rates reported in other countries. The five-year survival rate for STS overall in our country was 62.6% compared to 52–62.9% in other countries^6,8,25–27^. Although there are many subtypes for STS, the treatment modality has been similar, that is, wide resection with/without adjuvant radiotherapy^28^. A few types of sarcoma, including synovial sarcoma and myxoid/round cell liposarcoma, showed a significant improvement in survival after receiving adjuvant chemotherapy^29,30^. For GIST in Thailand, the 5-year survival rate was 63.4% compared to 63.6–67.7% in other countries^6,31^. The immunohistochemical staining of CD117 antigen (an epitope in the extracellular domain of c-kit) is an important confirmatory factor for GIST, and the positive staining of CD117 has been found in approximately 90% of GIST^32,33^. Surgery is the mainstay treatment for localized disease. Tyrosine kinase inhibitor (imatinib mesylate) is used for metastatic and inoperable GIST, based on the ESMO-EURACAN Clinical Practice Guidelines. This targeted therapy is an effective adjuvant treatment that improves the survival rate of GIST patients in Thailand similar to other studies in the post-imatinib era.

Because soft tissue sarcomas are a group of rare malignant tumors with high histological heterogeneity and a lack of exact molecular identification, the diagnosis is uncertain, even in high-standard countries^5–7^. Our country also has a similar limitation: the centrally second review by an expert pathologist is not commonly done in cancer registration. However, all the data collected from provinces that are at the center of the region were operated by university hospitals. Musculoskeletal pathologists and a tumor board performed all of the diagnoses. Additionally, for cancer registration system in Thailand, the tumor details and treatment data were not recorded as part of cancer registry. The standard protocol of clinical records has never been set across hospitals or nationwide protocol. This study provides an overview of the descriptive epidemiology of sarcomas in Thailand.

In summary, the annual incidence of STS and of GIST in Thailand analyzed here are approximately 2.14/100,000 person-years and 0.22/100,000 person-years, respectively, with an estimated new case annually in the country: 1604 of STS and 180 of GIST. The relatively low incidence of STS in Thailand and other Asian countries compared to Western countries underscores the association of race with the incidence of STS. Based on current healthcare service, an overall survival rates of STS and GIST are comparable to those reported from developed countries.

## Methods

### Study population

At present, Thailand has 11 cancer registries, all of which are operated by the Ministry of Public Health. This study included only the 5 which were population-based cancer registries (PBCRs) during the study period. The other 6 were excluded because their data was collected from hospital-based registries rather than population-based registries. Chiang Mai University (Chiang Mai province) launched the first PBCR in Thailand in 1986, followed by Khon Kaen University (Khon Kaen province) in 1988, Prince of Songkla University (Songkhla province) and the National Cancer Institute (Bangkok) in 1990, and the Lampang Cancer Hospital (Lampang province) in 1993. The five registries are located in each of the four major geographic areas of the country plus the capital city. The large majority of Thailand’s population are ethnic Thais, members of the East and Southeast Asian groupings that includes Chinese, Japanese and Koreans. Data was collected and analyzed retrospectively in a systematic manner. The data from the 5 PBCRs was either already coded by the PBCRs or was converted to codes by the research team according to the third edition of the International Classification of Diseases for Oncology (ICD-O-3). The morphology code indicating histological type and the topography code indicating primary site of STS and GIST were determined based on the 2002 and 2013 World Health Organization criteria (ICD-O-3: Morphology codes 8800–8935, 8910, 8920, 8940, 8950–8959, 8963–8964, 8990–8991, 9020–9044, 9120–9133, 9150, 9170, 9180, 9231, 9240, 9251, 9260, 9364–9372, 9540, 9560–9571, 9580–9581 combined with all ICD-O-3 Topography codes except C40.0–41.9 for STS, and Morphology codes 8936 combined with any Topography code for GIST)^4,6^. Data on sex, age at diagnosis, date of diagnosis, and basis of diagnosis were collected. The patient’s survival status was reviewed from Bureau of Registration Administration (BORA) database. The cancer registries data had been last updated on 31 December 2019.

Kaposi’s sarcoma (KS), virus-induced sarcoma, was excluded from the study because KS incidence patterns are strongly correlated with AIDS and thus would have introduced a major bias in the age and gender distribution of this sarcoma which could potentially have affected the present study^4^.

### Incidence analysis

STS and GIST data were reported as frequency, proportion, and incidence rate per 100,000 person-years. The incidence rates were calculated using population denominators from the Thailand National Census (Thailand National Statistical Office) and were categorized by sex (male and female), age group (18 groups: 0–4, 5–9, 10–14, 15–19, 20–24, 25–29, 30–34, 35–39, 40–44, 45–49, 50–54, 55–59, 60–64, 65–69, 70–74, 75–79, 80–84, 85 +), registry and histological type. The proportion of STS and GIST categorized by primary site was determined. The Segi world standard population^34^ was used to calculate age-standardized rates (ASRs). Age-specific and gender-specific incidence rates by histological types were plotted. The ASRs of females and males were compared using standardized rate ratios (SRRs). Time trend analysis was performed using Joinpoint Regression Program version 4.5.0.1. The trend and annual percent change (APC) in age-standardized incidence were calculated using Log-linear models. Time trends of STS, GIST and histological types over 5-year intervals were calculated.

### Survival analysis

All time-to-event analyses were performed using STATA program version 16.0. The Kaplan–Meier method was used to calculate patient survival rates. Five-year survival rates were determined and categorized by sex, histological type and primary site.

### Research ethics approval

The Research Ethics Committee 4, Faculty of Medicine, Chiang Mai University has approved this study protocol (Study code: ORT-2562-06377/ Research ID: 6377). All patients and/or parent provided informed consent for patient information to be stored in the hospital database. All methods were carried out in accordance with good clinical practices (GCPs) and relevant guidelines.

## Supplementary Information

Supplementary Information.

## Data Availability

All relevant data are included in this article and its supplementary material files, which are publicly available. Correspondence and requests for materials should be addressed to D.Pruksakorn.

## Abbreviations

PBCR: Population-based cancer registry
ASR: Age-standardized incidence rate
ICD-O-3: The third edition of the International Classification of Diseases for Oncology
SRR: Standardized rate ratios
APC: Annual percent change
MV: Morphological verification
DCO: Death certificate only verification
STS: Soft tissue sarcoma
GIST: Gastrointestinal stromal tumor
NOS: Not otherwise specified
UPS: Undifferentiated pleomorphic sarcoma
MFH: Malignant fibrous histiocytoma
MPNST: Malignant peripheral nerve sheath tumors

## References

[1] Fletcher CD, Unni KK, Mertens F (2002). Pathology and Genetics of Tumours of Soft Tissue and Bone.

[2] American Cancer Society. What is a soft tissue sarcoma? Available: https://www.cancer.org/cancer/soft-tissue-sarcoma/about/soft-tissue-sarcoma.html. (Accessed 28 June 2020).

[3] Fletcher C, Bridge JA, Hogendoorn PCW, Mertens F (2013). WHO Classification of Tumours of Soft Tissue and Bone: WHO Classification of Tumours.

[4] Amadeo B (2020). Incidence and time trends of sarcoma (2000–2013): Results from the French network of cancer registries (FRANCIM). BMC Cancer.

[5] Toro JR (2006). Incidence patterns of soft tissue sarcomas, regardless of primary site, in the surveillance, epidemiology and end results program, 1978–2001: An analysis of 26,758 cases. Int. J. Cancer..

[6] Stiller CA (2013). Descriptive epidemiology of sarcomas in Europe: Report from the RARECARE project. Eur. J. Cancer..

[7] Ducimetière F (2011). Incidence of sarcoma histotypes and molecular subtypes in a prospective epidemiological study with central pathology review and molecular testing. PLoS ONE.

[8] Trautmann F, Schuler M, Schmitt J (2015). Burden of soft-tissue and bone sarcoma in routine care: Estimation of incidence, prevalence and survival for health services research. Cancer Epidemiol..

[9] Mastrangelo G (2012). Incidence of soft tissue sarcoma and beyond: A population-based prospective study in 3 European regions. Cancer.

[10] Nomura E, Ioka A, Tsukuma H (2013). Incidence of soft tissue sarcoma focusing on gastrointestinal stromal sarcoma in Osaka, Japan, during 1978–2007. Jpn. J. Clin. Oncol..

[11] Hung G-Y (2019). Incidence of soft tissue sarcoma in Taiwan: A nationwide population-based study (2007–2013). Cancer Epidemiol..

[12] Stiller C, Parkint D (1994). International variations in the incidence of childhood soft-tissue sarcomas. Paediatr. Perinat. Ep..

[13] Jemal A (2006). Cancer statistics, 2006. CA-Cancer J. Clin..

[14] Sriamporn S (1996). Incidence of childhood cancer in Thailand 1988–1991. Paediatr. Perinat. Ep..

[15] Wiangnon S (2003). Childhood cancer in Thailand: 1995–1997. Asian Pac. J. Cancer.

[16] Wiangnon S (2011). Childhood cancer incidence and survival 2003–2005, Thailand: Study from the Thai Pediatric Oncology Group. Asian Pac. J. Cancer.

[17] Wiangnon S, Jetsrisuparb A, Komvilaisak P, Suwanrungruang K (2014). Childhood cancer incidence and survival 1985–2009, Khon Kaen. Thailand. Asian Pac. J. Cancer.

[18] Burningham Z, Hashibe M, Spector L, Schiffman JD (2012). The epidemiology of sarcoma. Clin. Sarcoma Res..

[19] Hsieh M-C, Wu X-C, Andrews PA, Chen VW (2013). Racial and ethnic disparities in the incidence and trends of soft tissue sarcoma among adolescents and young adults in the United States, 1995–2008. J. Adolesc. Young Adul..

[20] Kelley TW, Borden EC, Goldblum JR (2004). Estrogen and progesterone receptor expression in uterine and extrauterine leiomyosarcomas: an immunohistochemical study. Appl. Immunohisto. M. M..

[21] Jo VY, Fletcher CD (2014). WHO classification of soft tissue tumours: An update based on the 2013 (4th) edition. Pathology.

[22] Goettsch WG (2005). Incidence of gastrointestinal stromal tumours is underestimated: Results of a nation-wide study. Eur. J. Cancer..

[23] Lazar AJ, Trent JC, Lev D (2007). Sarcoma molecular testing: Diagnosis and prognosis. Curr. Oncol. Rep..

[24] Doyle LA (2014). Sarcoma classification: An update based on the 2013 World Health Organization Classification of Tumors of Soft Tissue and Bone. Cancer.

[25] Bessen T (2019). A population-based study of soft tissue sarcoma incidence and survival in Australia: an analysis of 26,970 cases. Cancer Epidemiol..

[26] Kollár A (2019). Incidence, mortality, and survival trends of soft tissue and bone sarcoma in Switzerland between 1996 and 2015. Cancer Epidemiol..

[27] Kim H-S, Han I (2021). Actual long-term survival after resection of stage III soft tissue sarcoma. BMC Cancer.

[28] Casali P (2018). Soft tissue and visceral sarcomas: ESMO–EURACAN Clinical Practice Guidelines for diagnosis, treatment and follow-up. Ann. Oncol..

[29] Karavasilis V (2008). Significant clinical benefit of first-line palliative chemotherapy in advanced soft-tissue sarcoma: Retrospective analysis and identification of prognostic factors in 488 patients. Cancer.

[30] Grimer R, Judson I, Peake D, Seddon B (2010). Guidelines for the management of soft tissue sarcomas. Sarcoma.

[31] Rubio J (2007). Population-based incidence and survival of gastrointestinal stromal tumours (GIST) in Girona, Spain. Eur. J. Cancer..

[32] Corless CL, McGreevey L, Haley A, Town A, Heinrich MC (2002). KIT mutations are common in incidental gastrointestinal stromal tumors one centimeter or less in size. Am. J. Pathol..

[33] Pornsuksiri K (2012). Clinical outcomes of gastrointestinal stromal tumor in southern Thailand. World J. Gastrointest. Oncol..

[34] Ahmad OB (2001). Age Standardization of Rates: A New WHO Standard.

