# Supplementary material for: Descriptive epidemiology of soft tissue sarcomas and gastrointestinal stromal tumors in Thailand
Source: Sci Rep. 2022 Jul 27;12:12824. doi: 10.1038/s41598-022-15711-8 (PMC9329344; doi:10.1038/s41598-022-15711-8)
Supplement: Supplementary file 1 — Supplementary Information. [file 41598_2022_15711_MOESM1_ESM.docx]

**Supplementary Materials**

**Descriptive epidemiology of soft tissue sarcomas and gastrointestinal stromal tumors in Thailand**

Jeerawan Klangjorhor, Donsuk Pongnikorn, Pattaralawan Sittiju, Areerak Phanphaisarn, Parunya Chaiyawat, Pimpisa Teeyakasem, Patiwat Kongdang, Sutpirat Moonmuang, Narate Waisri, Karnchana Daoprasert, Taweechok Wisanuyotin, Chalongpon Santong, Siriphon Sitthikong, Pakjai Tuntarattanapong, Paradee Prechawittayakul and Dumnoensun Pruksakorn

**
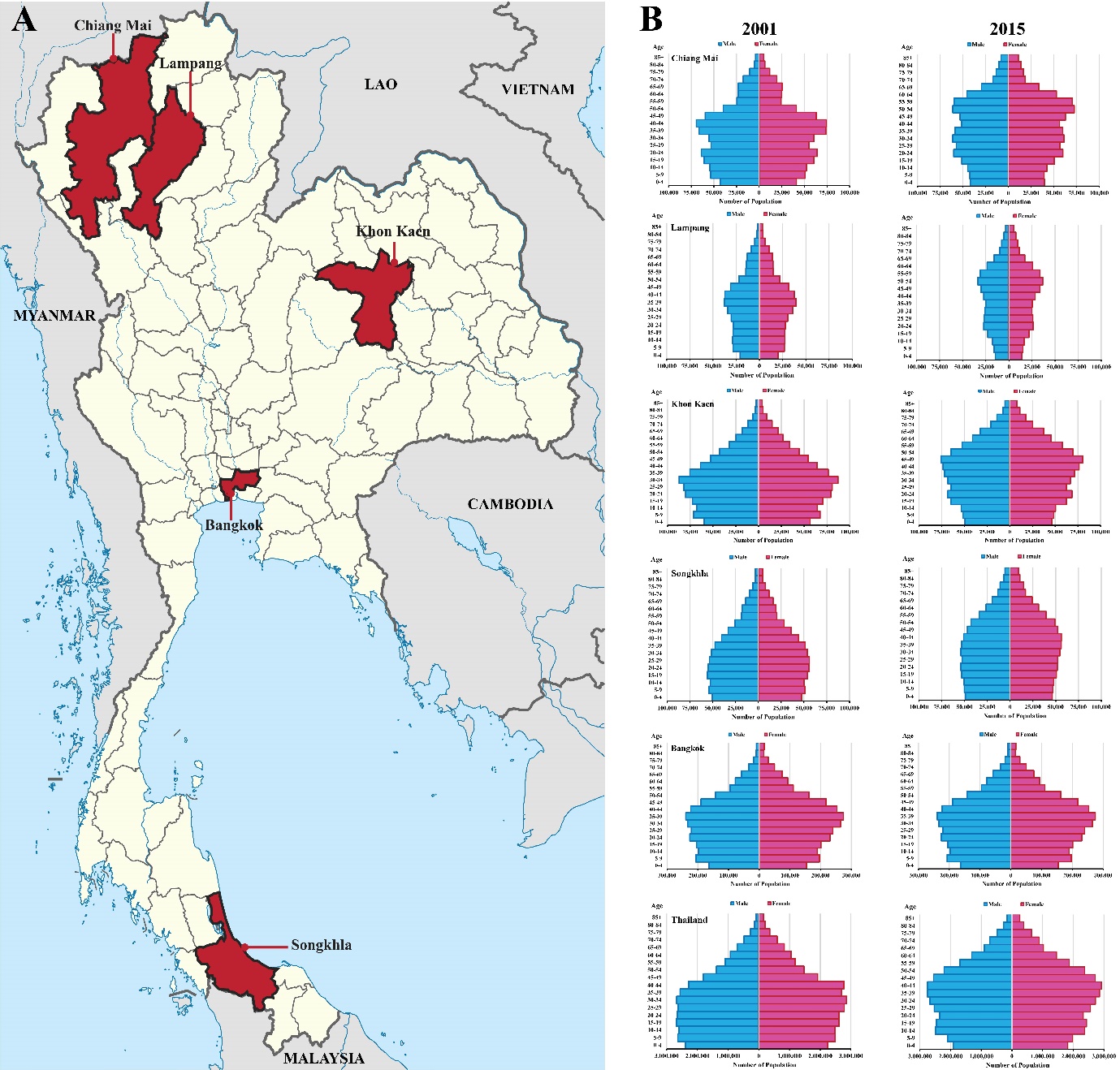
**

**Supplementary Figure S1.** Map of Thailand showing the five provincial population-based registries (A) and the population structure of each province (B) from which diagnosed STS and GIST cases during 2001-2015 were collected.


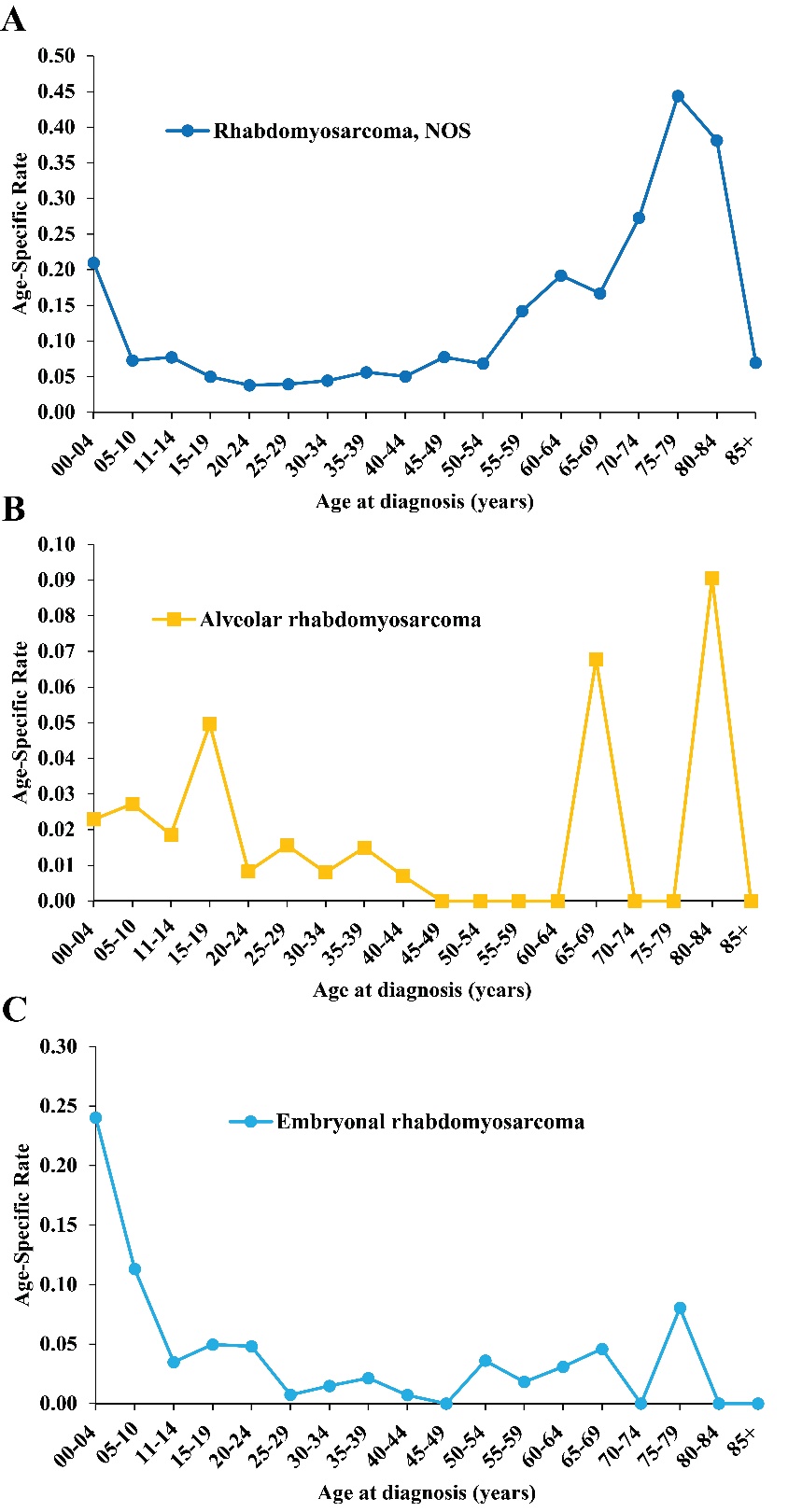


**Supplementary Figure S2.** Age-specific incidence rates of rhabdomyosarcoma by histological subtype.

**
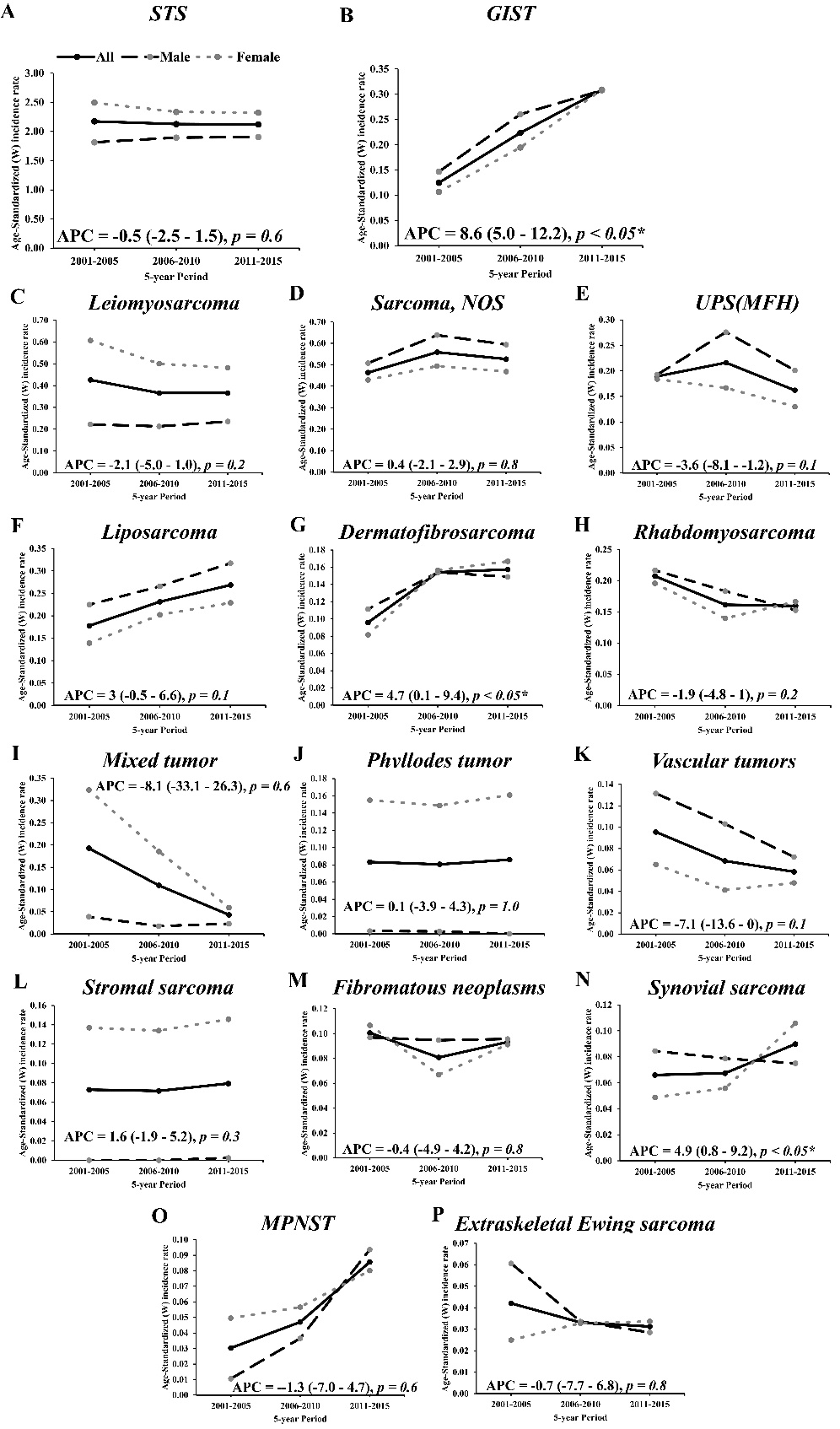
**

**Supplementary Figure S3.** Trends in age-standardized incidence rates of STS and GIST by major histological type and sex during 2001-2015. STS: soft tissue sarcoma; GIST: gastrointestinal stromal tumor; NOS: not otherwise specified; UPS: Undifferentiated pleomorphic sarcoma; MFH: malignant fibrous histiocytoma; MPNST: malignant peripheral nerve sheath tumors.

**
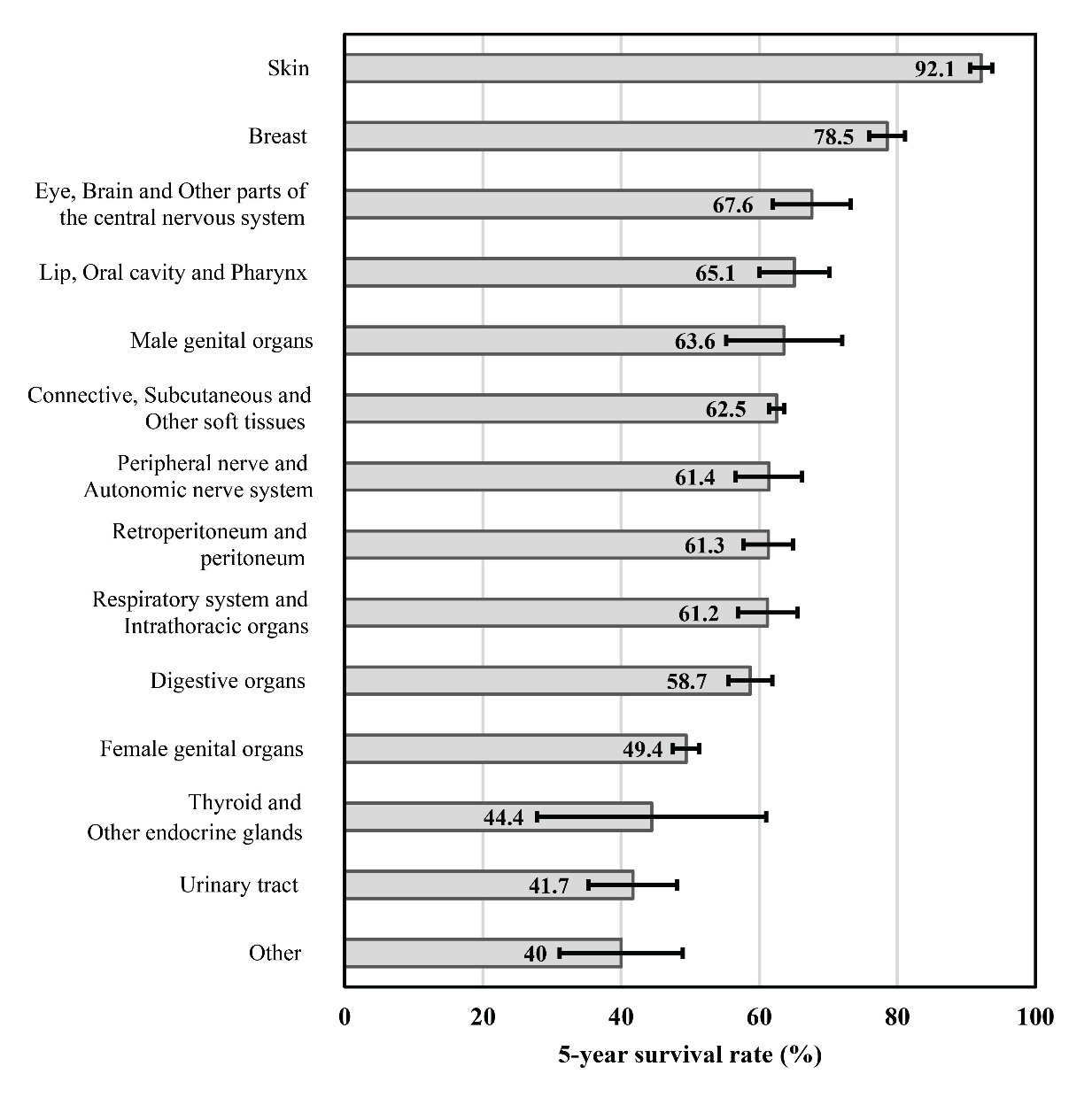
**

**Supplementary Figure S4.** Five-year observed survival rate of STS and GIST by primary site.


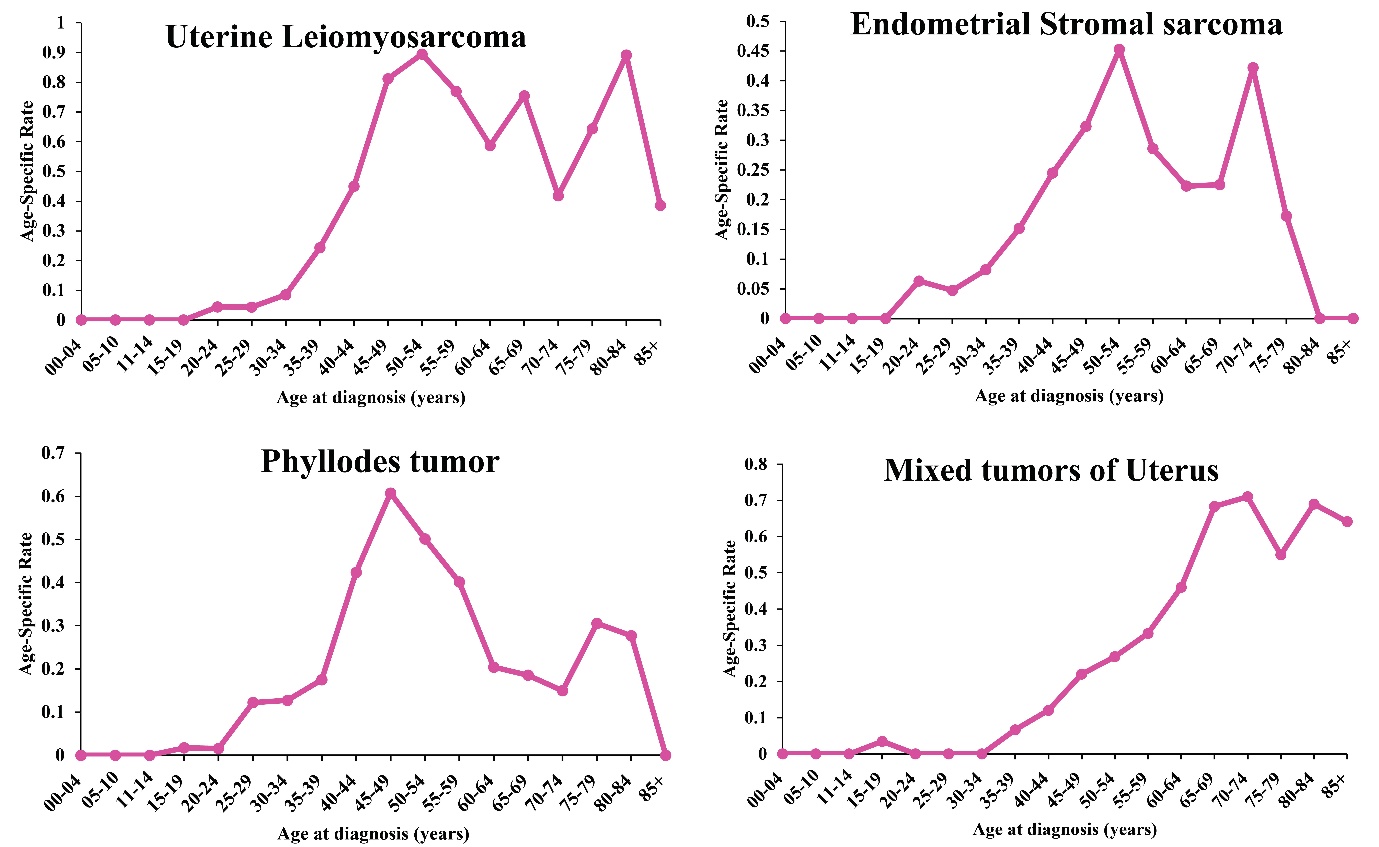


**Supplementary Figure S5**. Age-specific incidence rates of STS located in female genital organs. STS: soft tissue sarcoma.

**
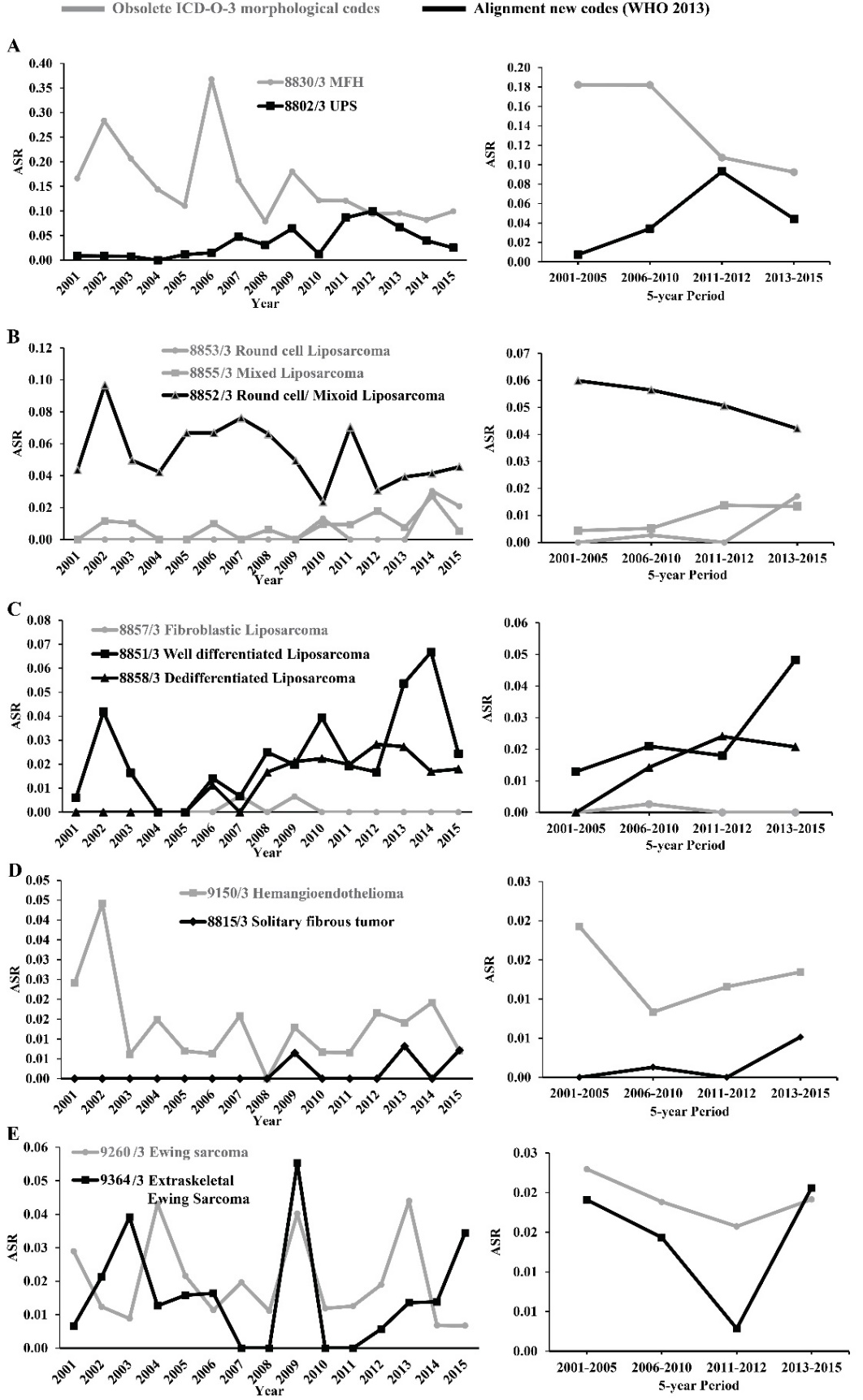
**

**Supplementary Figure S6.** List of obsolete ICD-O-3 morphological codes and their alignment with the 2013 World Health Organization (WHO) Classification of Tumors of Soft Tissue and Bone. ICD-O-3: the International Classification of Diseases for Oncology; MFH: malignant fibrous histiocytoma; UPS; undifferentiated pleomorphic sarcoma.

**
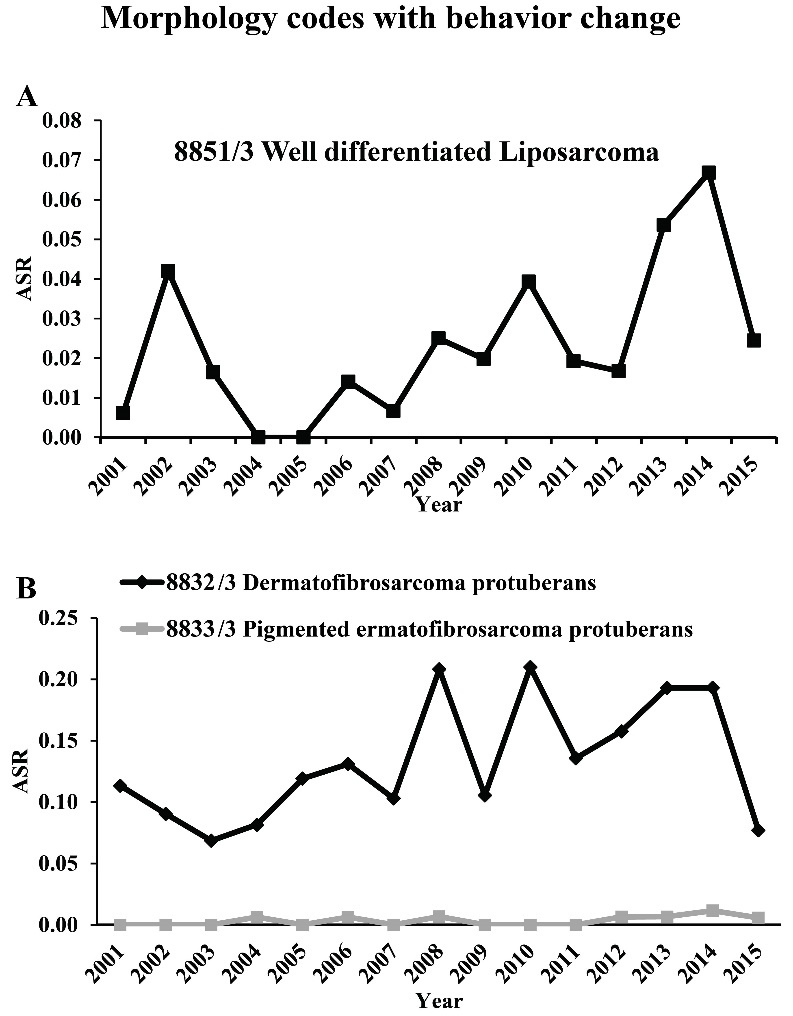
**

**Supplementary Figure S7.** List of terms of morphology whose behavior has been changed from "malignant" in ICD-O-3 to "borderline " in the 2013 World Health Organization (WHO) Classification of Tumors of Soft Tissue and Bone.

**Supplementary Table S1.** Number of cases, frequency, and data quality of soft tissue sarcoma cases collected from 5 PBCRs by basis of diagnosis

|  | **Cases** | **Percentage** | **%MV** | **%DCO** |
| --- | --- | --- | --- | --- |
| **Registry (*n* = 4080)** |  |  |  |  |
| Chiang Mai | 702 | 17.2 | 99.8 | 0.0 |
| Lampang | 235 | 5.8 | 100.0 | 0.0 |
| Khon Kaen | 459 | 11.3 | 99.1 | 0.0 |
| Bangkok | 2299 | 56.3 | 99.4 | 0.0 |
| Songkhla | 385 | 9.4 | 100.0 | 0.0 |
| **Sex (*n* = 4080)** |  |  |  |  |
| Male | 1634 | 40.0 | 99.6 | 0.0 |
| Female | 2446 | 60.0 | 99.5 | 0.0 |
| **Year of diagnosis (*n* = 4080)** |  |  |  |  |
| 2001-2005 | 1247 | 30.6 | 99.8 | 0.0 |
| 2006-2010 | 1344 | 32.9 | 99.8 | 0.0 |
| 2011-2015 | 1489 | 36.5 | 99.0 | 0.0 |
| **Age at diagnosis (years) (*n* = 4080)** |  |  |  |  |
| 0-9 | 116 | 2.8 | 99.1 | 0.0 |
| 10-19 | 177 | 4.3 | 100.0 | 0.0 |
| 20-29 | 283 | 6.9 | 99.3 | 0.0 |
| 30-39 | 512 | 12.5 | 99.6 | 0.0 |
| 40-49 | 803 | 19.7 | 99.2 | 0.0 |
| 50-59 | 947 | 23.2 | 99.7 | 0.0 |
| 60-69 | 641 | 15.7 | 99.4 | 0.0 |
| 70-79 | 415 | 10.2 | 99.8 | 0.0 |
| ≥ 80 | 186 | 4.6 | 100.0 | 0.0 |
| **Major type of soft tissue sarcoma (*n* = 4080)** |  |  |  |  |
| Sarcoma, NOS (8800-8806, 8830) | 1013 | 24.8 | 99.4 | 0.0 |
| UPS (MFH) (8830, 8802) | 379 | 9.3 | 100.0 | 0.0 |
| Leiomyosarcoma (8890, 8894-8896) | 775 | 19.0 | 99.5 | 0.0 |
| Liposarcoma (8850-8855, 8857-8858) | 464 | 11.4 | 100.0 | 0.0 |
| Dermatofibrosarcoma (8832, 8833) | 263 | 6.4 | 99.2 | 0.0 |
| Rhabdomyosarcoma (8900-8902, 8910, 8912, 8920) | 241 | 5.9 | 99.6 | 0.0 |
| Mixed tumors (8940, 8950-8951) | 213 | 5.2 | 100.0 | 0.0 |
| Phyllodes tumor (9020) | 178 | 4.4 | 99.4 | 0.0 |
| Fibromatous neoplasms (8810-8811, 8814-8815, 9150) | 176 | 4.3 | 100.0 | 0.0 |
| MPNST (9540, 9560, 9561) | 165 | 4.0 | 100.0 | 0.0 |
| Stromal sarcoma (8930,8931,8933,8935) | 155 | 3.8 | 98.1 | 0.0 |
| Stromal sarcoma (8930,8931,8933,8935) | 155 | 3.8 | 98.1 | 0.0 |
| Vascular tumors (9120, 9130, 9133) | 147 | 3.6 | 100.0 | 0.0 |
| Synovial sarcoma (9040-9041, 9043-9044) | 134 | 3.3 | 99.2 | 0.0 |
| Extraskeletal Ewing sarcoma (9260, 9364) | 50 | 1.2 | 98.0 | 0.0 |
| Extra-renal rhabdoid tumor (8963, 8964) | 19 | 0.5 | 100.0 | 0.0 |
| Granular cell tumor (9580) | 18 | 0.4 | 100.0 | 0.0 |
| Alveolar soft part sarcoma (9581) | 17 | 0.4 | 100.0 | 0.0 |
| Phosphaturic mesenchymal tumor (8990, 8991) | 13 | 0.3 | 100.0 | 0.0 |
| Extraskeletal chondrosarcoma (9231,9240) | 12 | 0.3 | 100.0 | 0.0 |
| Chordoma (9370, 9371) | 11 | 0.3 | 100.0 | 0.0 |
| Myxosarcoma (8840) | 7 | 0.2 | 100.0 | 0.0 |
| Perineurioma (9571) | 4 | 0.1 | 100.0 | 0.0 |
| Giant cell sarcoma of soft parts (9251) | 3 | 0.1 | 100.0 | 0.0 |
| Myofibroblastic sarcoma (8825) | 2 | 0.05 | 100.0 | 0.0 |

PBCR: Population-based cancer registry; %MV: percentage of morphologically verified cases; %DCO: percentage of cases verified by death certificate only; NOS: Not otherwise specified; MFH: Malignant fibrous histiocytoma; UPS: Undifferentiated pleomorphic sarcoma; MPNST: Malignant peripheral nerve sheath tumors.

**Supplementary Table S2.** Number of cases, frequency, and data quality of gastrointestinal stromal tumor cases collected from 5 PBCRs by basis of diagnosis

|  | **Cases** | **Percentage** | **%MV** | **%DCO** |
| --- | --- | --- | --- | --- |
| **Registry (*n* = 457)** |  |  |  |  |
| Chiang Mai | 77 | 16.8 | 100.0 | 0.0 |
| Lampang | 23 | 5.3 | 100.0 | 0.0 |
| Khon Kaen | 57 | 12.5 | 98.2 | 0.0 |
| Bangkok | 291 | 63.7 | 98.6 | 0.0 |
| Songkhla | 9 | 2.1 | 100.0 | 0.0 |
| **Sex (*n* = 457)** |  |  |  |  |
| Male | 223 | 48.8 | 98.6 | 0.0 |
| Female | 234 | 51.2 | 99.6 | 0.0 |
| **Year of diagnosis (*n* = 457)** |  |  |  |  |
| 2001-2005 | 72 | 15.8 | 100.0 | 0.0 |
| 2006-2010 | 150 | 32.8 | 99.3 | 0.0 |
| 2011-2015 | 235 | 51.4 | 98.3 | 0.0 |
| **Age at diagnosis (years) (*n* = 457)** |  |  |  |  |
| 0-9 | 0 | 0.0 | 0.0 | 0.0 |
| 10-19 | 5 | 1.1 | 100.0 | 0.0 |
| 20-29 | 9 | 2.0 | 100.0 | 0.0 |
| 30-39 | 28 | 6.1 | 96.4 | 0.0 |
| 40-49 | 63 | 13.8 | 98.4 | 0.0 |
| 50-59 | 117 | 25.6 | 100.0 | 0.0 |
| 60-69 | 129 | 28.2 | 99.2 | 0.0 |
| 70-79 | 81 | 17.7 | 97.5 | 0.0 |
| ≥ 80 | 25 | 5.5 | 100.0 | 0.0 |

PBCR: Population-based cancer registry; %MV: percentage of morphologically verified cases; %DCO: percentage of cases verified by death certificate only.

**Supplementary Table S3.** Soft tissue sarcomas and gastrointestinal stromal sarcoma rate by anatomic sites and sex diagnosed during 2001-2015 in Thailand

| **Topography** | | | **All** | | **Male** | | **Female** | |
| --- | --- | --- | --- | --- | --- | --- | --- | --- |
|  |  |  | ***n*** | ***%*** | ***n*** | ***%*** | ***n*** | ***%*** |
|  | | |  |  |  |  |  |  |
| **Soft tissue sarcoma** | | | 4,080 | 100 | 1634 | 40.0 | 2446 | 60.0 |
|  | C49 | Connective, Subcutaneous and Other soft tissues | 1866 | 45.7 | 974 | 23.9 | 892 | 21.9 |
|  | C51-C57 | Female genital organs | 747 | 18.3 | 0 | 0.0 | 747 | 18.3 |
|  | C44 | Skin | 281 | 6.9 | 144 | 3.5 | 137 | 3.4 |
|  | C15-C26 | Digestive organs | 238 | 5.8 | 137 | 3.4 | 101 | 2.5 |
|  | C50 | Breast | 244 | 6.0 | 3 | 0.1 | 241 | 5.9 |
|  | C48 | Retroperitoneum and peritoneum | 181 | 4.4 | 86 | 2.1 | 95 | 2.3 |
|  | C30-C39 | Respiratory system and Intrathoracic organs | 130 | 3.2 | 66 | 1.6 | 64 | 1.6 |
|  | C47 | Peripheral nerve and Autonomic nerve system | 102 | 2.5 | 48 | 1.2 | 54 | 1.3 |
|  | C00-C14 | Lip, Oral cavity and Pharynx | 86 | 2.1 | 51 | 1.3 | 35 | 0.9 |
|  | C69-C72 | Eye, Brain and Other parts of the central nervous system | 72 | 1.8 | 35 | 0.9 | 37 | 0.9 |
|  | C64-C68 | Urinary tract | 61 | 1.5 | 33 | 0.8 | 28 | 0.7 |
|  | C60-C63 | Male genital organs | 33 | 0.8 | 33 | 0.8 | 0 | 0.0 |
|  | C73-C75 | Thyroid and Other endocrine glands | 9 | 0.2 | 4 | 0.1 | 5 | 0.1 |
|  | C76, C77, C80 | Other, Ill-defined sited, Lymph nodes and Unknown primary site | 30 | 0.7 | 20 | 0.5 | 10 | 0.2 |
|  |  |  |  |  |  |  |  |  |
| **Gastrointestinal Stromal tumor** | | | 457 | 100 | 223 | 48.8 | 234 | 51.2 |
|  | C16 | Stomach | 209 | 45.7 | 95 | 20.8 | 114 | 24.9 |
|  | C17 | Small intestine | 113 | 24.7 | 57 | 12.5 | 46 | 12.3 |
|  | C20 | Rectum | 35 | 7.7 | 22 | 4.8 | 13 | 2.8 |
|  | C18 | Colon | 32 | 7.0 | 19 | 4.2 | 13 | 2.8 |
|  | C19 | Rectosigmoid junction | 11 | 2.4 | 4 | 0.9 | 7 | 1.5 |
|  | C15 | Esophagus | 4 | 0.9 | 1 | 0.2 | 3 | 0.7 |
|  | C21 | Anal canal and anus | 1 | 0.2 | 1 | 0.2 | 0 | 0.0 |
|  | C23 | Gallbladder | 1 | 0.2 | 1 | 0.2 | 0 | 0.0 |
|  | C25 | Pancreas | 1 | 0.2 | 0 | 0.0 | 1 | 0.2 |
|  | C26 | Unspecified digest. organs | 50 | 10.9 | 23 | 5.0 | 27 | 5.9 |
|  |  |  |  |  |  |  |  |  |

**Supplementary Table S4.** Occurrence by histological types of soft tissue sarcoma by anatomic site

| **Topography** | | | **Leiomyosarcoma** | | **Sarcoma, NOS** | | **UPS (MFH)** | | **Liposarcoma** | | **Dermatofibrosarcoma** | | **Rhabdomyosarcoma** | | **Mixed tumor** | | **Phyllodes tumor** | | **Vascular tumors** | | **Stromal sarcoma** | | **Fibromatous neoplasms** | | **Synovial sarcoma** | | **MPNST** | |
| --- | --- | --- | --- | --- | --- | --- | --- | --- | --- | --- | --- | --- | --- | --- | --- | --- | --- | --- | --- | --- | --- | --- | --- | --- | --- | --- | --- | --- |
|  |  |  | **n** | **%** | **n** | **%** |  |  | **n** | **%** | **n** | **%** | **n** | **%** | **n** | **%** | **n** | **%** | **n** | **%** | **n** | **%** | **n** | **%** | **n** | **%** | **n** | **%** |
| **All primary site** | | | 775 | 100 | 1013 | 100 | 379 | 100 | 464 | 100 | 263 | 100 | 241 | 100 | 213 | 100 | 178 | 100 | 147 | 100 | 155 | 100 | 176 | 100 | 134 | 100 | 165 | 100 |
|  | C49 | Connective, Subcutaneous and Other soft tissues | 257 | 33.2 | 684 | 67.5 | 303 | 79.9 | 345 | 74.4 | 10 | 3.8 | 136 | 56.4 | 0 | 0.0 | 0 | 0.0 | 55 | 37.4 | 2 | 1.3 | 137 | 77.8 | 118 | 88.1 | 44 | 26.7 |
|  | C51-C57 | Female genital organs | 311 | 40.1 | 57 | 5.6 | 5 | 1.3 | 4 | 0.9 | 1 | 0.4 | 19 | 7.9 | 180 | 84.5 | 0 | 0.0 | 0 | 0.0 | 150 | 96.8 | 7 | 4.0 | 0 | 0.0 | 1 | 0.6 |
|  | C44 | Skin | 4 | 0.5 | 11 | 1.1 | 4 | 1.1 | 4 | 0.9 | 252 | 95.8 | 2 | 0.8 | 2 | 0.9 | 0 | 0.0 | 2 | 1.4 |  | 0.0 | 3 | 1.7 | 0 | 0.0 | 0 | 0.0 |
|  | C15-C26 | Digestive organs | 89 | 11.5 | 60 | 5.9 | 7 | 1.8 | 9 | 1.9 | 0 | 0.0 | 1 | 0.4 | 2 | 0.9 | 0 | 0.0 | 61 | 41.5 | 1 | 0.6 | 4 | 2.3 | 1 | 0.7 | 1 | 0.6 |
|  | C50 | Breast | 1 | 0.1 | 36 | 3.6 | 10 | 2.6 | 6 | 1.3 | 0 | 0.0 | 6 | 2.5 | 0 | 0.0 | 178 | 100 | 13 | 8.8 | 1 | 0.6 | 1 | 0.6 | 0 | 0.0 | 0 | 0.0 |
|  | C48 | Retroperitoneum and peritoneum | 54 | 7.0 | 38 | 3.8 | 17 | 4.5 | 66 | 14.2 | 0 | 0.0 | 8 | 3.3 | 0 | 0.0 | 0 | 0.0 | 0 | 0.0 | 0 | 0.0 | 3 | 1.7 | 5 | 3.7 | 6 | 3.6 |
|  | C30-C39 | Respiratory system and Intrathoracic organs | 10 | 1.3 | 60 | 5.9 | 15 | 4.0 | 8 | 1.7 | 0 | 0.0 | 24 | 10.0 | 0 | 0.0 | 0 | 0.0 | 6 | 4.1 | 0 | 0.0 | 6 | 3.4 | 5 | 3.7 | 3 | 1.8 |
|  | C47 | Peripheral nerve and Autonomic nerve system | 2 | 0.3 | 12 | 1.2 | 6 | 1.6 | 3 | 0.6 | 0 | 0.0 | 0 | 0.0 | 0 | 0.0 | 0 | 0.0 | 0 | 0.0 | 0 | 0.0 | 1 | 0.6 | 0 | 0.0 | 77 | 46.7 |
|  | C00-C14 | Lip, Oral cavity and Pharynx | 14 | 1.8 | 13 | 1.3 | 3 | 0.8 | 8 | 1.7 | 0 | 0.0 | 9 | 3.7 | 24 | 11.3 | 0 | 0.0 | 8 | 5.4 | 0 | 0.0 | 3 | 1.7 | 1 | 0.7 | 1 | 0.6 |
|  | C69-C72 | Eye, Brain and  Other parts of the central nervous system | 0 | 0.0 | 11 | 1.1 | 1 | 0.3 | 1 | 0.2 | 0 | 0.0 | 9 | 3.7 | 1 | 0.5 | 0 | 0.0 | 1 | 0.7 | 0 | 0.0 | 9 | 5.1 | 1 | 0.7 | 28 | 17.0 |
|  | C64-C68 | Urinary tract | 15 | 1.9 | 19 | 1.9 | 4 | 1.1 | 2 | 0.4 | 0 | 0.0 | 11 | 4.6 | 0 | 0.0 | 0 | 0.0 | 0 | 0.0 | 0 | 0.0 | 1 | 0.6 | 2 | 1.5 | 2 | 1.2 |
|  | C60-C63 | Male genital organs | 6 | 0.8 | 3 | 0.3 | 0 | 0.0 | 7 | 1.5 | 0 | 0.0 | 12 | 5.0 | 1 | 0.5 | 0 | 0.0 | 0 | 0.0 | 1 | 0.6 | 0 | 0.0 | 0 | 0.0 | 0 | 0.0 |
|  | C73-C75 | Thyroid and Other endocrine glands | 5 | 0.6 | 1 | 0.1 | 1 | 0.3 | 0 | 0.0 | 0 | 0.0 | 1 | 0.4 | 0 | 0.0 | 0 | 0.0 | 0 | 0.0 | 0 | 0.0 | 0 | 0.0 | 0 | 0.0 | 1 | 0.6 |
|  | C76, C77, C80 | Other, Ill-defined sited, Lymph nodes  and Unknown primary site | 7 | 0.9 | 8 | 0.8 | 3 | 0.8 | 1 | 0.2 | 0 | 0.0 | 3 | 1.2 | 3 | 1.4 | 0 | 0.0 | 1 | 0.7 | 0 | 0.0 | 1 | 0.6 | 1 | 0.7 | 1 | 0.6 |

NOS: not otherwise specified; UPS: Undifferentiated pleomorphic sarcoma; MFH: malignant fibrous histiocytoma; MPNST: malignant peripheral nerve sheath tumors.
